# *NYD-OP7/PLC* regulatory signaling pathway regulates deltamethrin resistance in *Culex pipiens pallens* (Diptera: Culicidae)

**DOI:** 10.1186/s13071-018-3011-5

**Published:** 2018-07-16

**Authors:** Dan Zhou, Baiyun Duan, Yang Xu, Lei Ma, Bo Shen, Yan Sun, Changliang Zhu

**Affiliations:** Department of Pathogen Biology, Nanjing Medical University, Nanjing, China

**Keywords:** *Culex pipiens pallens*, Insecticide resistance, Deltamethrin, Opsin, Phospholipase C

## Abstract

**Background:**

Investigation of insecticide resistance mechanisms is considered a vital first step towards the creation of effective strategies to control resistant mosquitoes and manage mosquito-borne diseases. Our previous study revealed that NYD-OP7 may be associated with deltamethrin resistance in *Culex pipiens pallen*. However, the precise function of NYD-OP7 in deltamethrin resistance is still unclear. In this study, we investigated the role of NYD-OP7 in the molecular mechanisms underlying pyrethroid resistance.

**Results:**

Knockdown of *NYD-OP7* not only increased the susceptibility of the mosquitoes to deltamethrin *in vivo* but also simultaneously repressed both expression and enzyme activity of its downstream effector molecule, *phospholipase C* (*PLC*) and expression of several insecticide resistance-related P450 genes. Knockdown of *PLC* also sensitized the mosquitoes to deltamethrin and reduced the expression of the P450 genes.

**Conclusions:**

Our results revealed that *NYD-OP7* and its downstream effector *PLC* contribute to deltamethrin resistance by regulating the expression of P450s in *Cx. pipiens pallens*.

**Electronic supplementary material:**

The online version of this article (10.1186/s13071-018-3011-5) contains supplementary material, which is available to authorized users.

## Background

Mosquitoes can transmit numerous serious infectious diseases, such as malaria, dengue fever, Zika, West Nile fever, chikungunya, yellow fever, Rift Valley fever, La Crosse encephalitis, Japanese encephalitis and filariasis [1–10]. Chemical insecticides are one of the mainstay strategies for the control of mosquito vectors. Unfortunately, the heavy reliance on pesticide has led to the development of resistance in vectors, making insecticide use ineffective [11]. Resistance to insecticides has been reported in many mosquito species, implicating a major obstacle for the control of vector-borne diseases [12–14].

Many studies have indicated that insecticide resistance is actually a complex phenotype of polygenic inheritance phenomenon [15–19]. To date, three major mechanisms are responsible for insecticide resistance in mosquitoes: alterations in the target sites, increased metabolic detoxification, and reduced cuticular penetration [20–22]. In addition, many genes that are not associated with the above-mentioned mechanisms may be involved in insecticide resistance. For example, several *Anopheles gambiae* genes, such as sodium/calcium exchanger, peptidases, and genes responsible for lipid and carbohydrate metabolism, were highly expressed in an insecticide-resistant strain [16]. The upregulation of protease genes were also observed in pyrethroid resistant *Cx. quinquefasciatus* [23]. A previous study in our laboratory also identified several differentially expressed genes, such as arrestin, glycogen branching enzyme or prophenoloxidase gene, between the deltamethrin-susceptible (DS) strain and deltamethrin-resistant (DR) strain in *Cx. pipiens pallens* [24–26]. However, the potential roles of these genes in the development of the resistance phenotype are unclear. Further functional characterizations are needed to help in understanding the relationships between these genes and resistance processes.

Opsins, which belong to the G-protein-coupled receptor (GPCR) superfamily, are primarily involved in the visual signaling cascade [27]. In invertebrates, activation of the downstream effector molecule phospholipase C (PLC) is critical in the phototransduction cascades [28]. Previous studies have indicated that GPCR members have broader and more diverse functions than as light sensors in animals. For example, *OPN3* is an asthma susceptibility gene that plays a role in immune modulation [29]. Rhodopsin may serve as the ATP-independent phospholipid flippase [30]. UV-sensitive and blue color-sensitive opsins were found to be overexpressed in DDT-resistant *Drosophila*, suggesting opsins may be associated with insecticide resistance [31]. The GPCR signaling pathway can regulate resistance-related P450 genes in *Cx. quinquefasciatus* [32].

In our previous study, suppression subtractive hybridization (SSH) analysis revealed that *NYD-OP7* (GenBank: AY749413), which belongs to the invertebrate Gq-coupled opsin subfamily, was overexpressed at the transcriptional level in the laboratory-selected DR strain of *Cx. pipiens pallens* [33]. Furthermore, the overexpression of *NYD-OP7* increased the resistance of *Aedes albopictus* C6/36 cells to deltamethrin *in vitro* [34]. Although these results suggest *NYD-OP7* is associated with deltamethrin resistance, there are still many unanswered questions regarding the functions of *NYD-OP7* in the development of the resistance. In particular, the mechanism underlying the regulation of insecticide resistance by *NYD-OP7* needs to be elucidated.

In this study, the expression levels of *NYD-OP7* in the laboratory DS and DR strains of *Cx. pipiens pallens* were detected using western blotting, and our previous SSH and quantitative real-time (qPCR) analyses results were validated. The roles of *NYD-OP7* and its downstream effector molecule *PLC* on insecticide resistance were also preliminarily investigated *in vivo*.

## Methods

### Mosquito strains

In this study, two laboratory strains of *Cx. pipiens pallens* were used. In 2010, the DS strain [50% lethal concentration (LC_50_) = 0.01 ppm] was obtained from the Jiangsu Institute of Parasitic Diseases in Wuxi (Jiangsu Province, China) and then maintained in our laboratory without exposure to any insecticides. The DR strain was selected from the early fourth-instar larvae of the DS strain with deltamethrin. Before selection, LC_50_ was determined using a larval bioassay and then used as the selection concentration. Finally, 58 generations of the DR strain were reared with an LC_50_ of 7.3 ppm. The mosquitoes were maintained at 28 °C, 70–80% relative humidity, and a constant light/dark photoperiod (16/8 h).

### RNA extraction and cDNA synthesis

Total RNA was extracted from 5 female mosquitoes from each group by using TRIzol reagent (Invitrogen, Carlsbad, CA, USA), according to the manufacturer’s protocol. The integrity of the isolated total RNA was assessed using 1% agarose gel electrophoresis. The purity and concentration were checked with a spectrophotometer (NanoDrop, Wilmington, DE, USA). The RNA was used for cDNA synthesis only if the gel electrophoresis showed clear bands of 28S and 18S and the ratio of OD260/OD280 was within the range between 1.8 and 2.0 [35, 36]. cDNA was synthesized from 500 ng of total RNA by using the PrimeScriptRT Reagent Kit (TaKaRa, Tokyo, Japan), according to the manufacturer’s protocol.

### qPCR analysis

qPCR was performed using the LightCycler® 96 Instrument (Roche, Basel, Switzerland) with Power SYBR Green PCR Master Mix (Applied Biosystems, Foster, USA), according to the manufacturer’s protocol. The reaction volume (20 μl) contained the Power SYBR Green PCR Master Mix, specific forward and reverse primers (Additional file 1: Table S1) and diluted cDNA. The PCR conditions were as follows: 50 °C for 2 min and 95 °C for 10 min, followed by 40 cycles at 95 °C for 15 s and 60 °C for 1 min. Both melting curve analysis and gel electrophoresis of the amplification products were performed to confirm that the primers amplified only a single product of the expected size. In addition, the qPCR products were sequenced for confirmation. The raw threshold cycle (Ct) values were used to quantify the target gene expression for each sample. The relative expression levels were normalized to the internal control *β-actin* by using the 2^−ΔΔCt^ method [37]: target gene /*β-actin* = 2^ΔCt^, ΔCt = Ct_β-actin_ − Ct_target gene_. Three technical and biological replicates were performed for qPCR analyses.

### Preparation of NYD-OP7 antibody

The amino acid sequence of NYD-OP7 was submitted to the BepiPred 1.0 server (http://www.cbs.dtu.dk/services/BepiPred/), and the epitope (CVASGATTASDEKA) was predicted. The peptide of 14 amino acids was chemically synthesized and used as an immunogen to immunize 2 female New Zealand white rabbits. Before inoculation, the 2 rabbits were bled to obtain 30 to 50 ml of preimmune serum as the negative control. The primary immunization consisted of 1000 μl of the immunogen [1 μg/μl, dissolved in phosphate-buffered saline (PBS)] mixed with an equal volume of Freund’s complete adjuvant. For the subsequent immunizations, 500 μl of the immunogen (1 μg/μl, dissolved in PBS) was mixed with an equal volume of Freund’s incomplete adjuvant. After 4 immunizations, the antiserum was harvested and subjected to affinity purification (SAB Biotech, Nanjing, China). The sensitivity of the developed NYD-OP7 antibody was measured by enzyme-linked immunosorbent assay (ELISA, ELISA > 1:128,000). We checked the specificity of the antibody by western blot, which showed only one band and the same molecular weight as the target protein.

### Western blot analysis

Proteins were extracted from the DS and DR strains of *Cx. pipiens pallens* with the RIPA lysis buffer (Beyotime, Shanghai, China) containing the protease inhibitor PMSF, according to the manufacturer’s instructions. The concentrations were determined using the BCA Protein Assay Kit (Pierce, Rockford, USA). Up to 40 μg of protein per lane was used for performing sodium dodecyl sulfate-polyacrylamide gel electrophoresis (SDS-PAGE) with 12% gels. SDS-PAGE was performed at 80 V for 30 min and 120 V for 80 min. The proteins were then transferred to a polyvinylidene fluoride membrane for 40 min at 300 mA by using the Trans-Blot SD Cell and Systems (Bio-Rad, Hercules, CA, USA). NYD-OP7 was detected using the prepared polyclonal antibody (1:1000) at 4 °C overnight and horseradish peroxidase–conjugated goat anti-rabbit secondary antibody (1:2000, Beyotime) for 2 h at 28 °C. The anti-tubulin monoclonal antibody (1:1000; CW Biotech, Beijing, China) was used as the internal control [24]. Chemiluminescence was detected using BeyoECL Plus (Pierce), according to the manufacturer’s instructions.

### Microinjection

The dsRNA of *NYD-OP7* (dsNYD-OP7), siRNA of *PLC* (siPLC), and negative control (NC) were designed and synthesized by GenePharma (Shanghai, China; Additional file 2: Table S2). NC is a sequence generated from a nematode which showed no homology compared to mosquitoes. Two controls were used: an equivalent volume of DEPC water or NC. The microinjection experiment was performed using day 1 post-emergence female mosquitoes. About 360 ng of dsNYD-OP7, 364 ng of siPLC, or 350 ng of NC were injected into the side of the protocoel of the female mosquitoes with a microinjector (Drummond’s Nanoject II, # 3-000-205A, Pennsylvania, USA) attached to 3.5" needles (Drummond Scientific Company, # 3-000-203-G/X). Then, the mosquitoes were maintained in the insectary at 28 °C and a constant light/dark photoperiod (16/8 h) with 70–80% relative humidity. Three days post-injection, gene silencing efficiency on random selected mosquitoes was determined using qPCR, and the remaining mosquitoes were then selected for subsequent experiments.

### CDC bottle bioassay

The resistance of the mosquitoes to insecticides was detected using the CDC bottle bioassay. The diagnostic dose used in the present study was determined using the calibration assay (http://www.cdc.gov/parasites/education_training/lab/bottlebioassay.html). In each bottle, 20 adult female mosquitoes were exposed to deltamethrin, and a bottle coated with acetone was used as the control. The final concentration of deltamethrin was 7.5 mg/ml. The numbers of dead and alive mosquitoes were recorded at 15 min intervals for 2 h (15, 30, 45, 60, 75, 90, 105 and 120 min) or until all the mosquitoes died. The mosquitoes were considered dead if they could not fly or maintain an upright posture on the surface of the bottle. The total percentage mortality against time was calculated for all the replicates. The experiment was repeated 3 times.

### PLC enzyme activity assay

PLC enzyme activity was analyzed in the dsNYD-OP7, siPLC, NC, and DEPC groups. Non-blood-fed female adult mosquitoes were rinsed 3 times with ddH_2_O to remove food particles and molted skin. PLC enzyme activity of the mosquitoes was detected using the tissue phospholipase C activity of continuous circulation colorimetric assay kit (Genmed Scientifics Inc., Wilmington, USA), according to the manufacturer’s protocol. The protein content in the supernatant was measured using the Enhanced BCA Protein Assay Kit (Beyotime). Each group was composed of 25 mosquitoes, and all the assays were performed in duplicate.

### Statistical analysis

The data were analyzed using Student’s t-test and Chi-square test. A *P*-value < 0.05 was considered statistically significant. All results were presented as mean and SEM values of 3 independent experiments.

## Results

### NYD-OP7 is differentially expressed in a DR strain of *Cx. pipiens pallens*

**Fig. 1:**
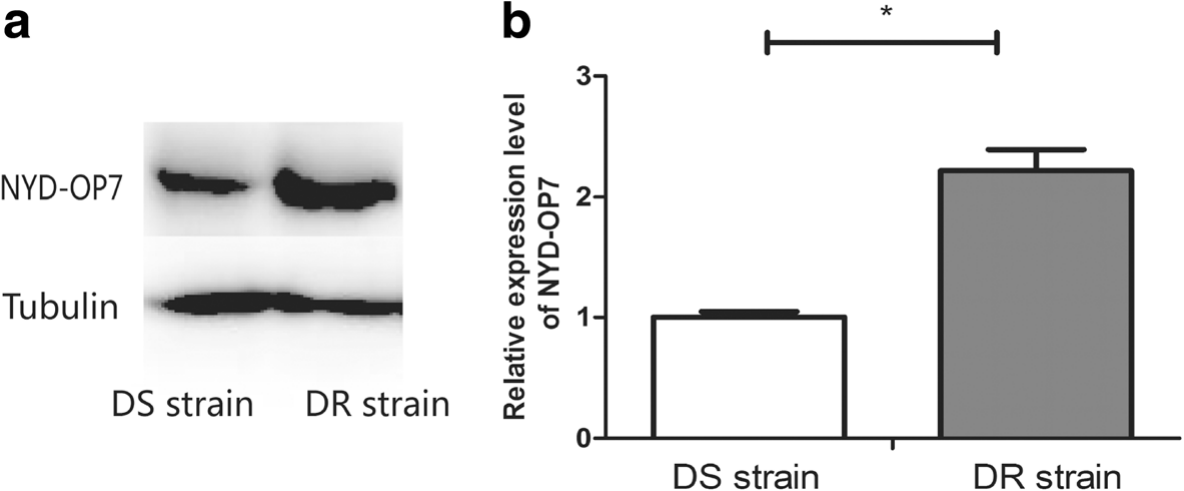
Identification of NYD-OP7 in the deltamethrin-susceptible (DS) and deltamethrin-resistant (DR) strains of *Culex pipiens pallens*. **a** Western blot analysis of NYD-OP7 in the DS and DR strains of *Cx. pipiens pallens*. The amounts of samples loaded were monitored by determining tubulin. **b** NYD-OP7 expression in the DR strain was about 2.2-fold higher than that in the DS strain at the protein level. The intensity of the protein band was normalized with tubulin band intensity in each corresponding lane

Expression of NYD-OP7 in the DS and DR strains of *Cx. pipiens pallens* were analyzed using western blotting. The level of NYD-OP7 expression in the DR strain was 2.2-fold higher than that in the DS strain (Fig. 1a, b). This finding further strengthened the evidence linking the overexpression of NYD-OP7 with deltamethrin resistance.

### Role of *NYD-OP7* in deltamethrin resistance

**Fig. 2:**
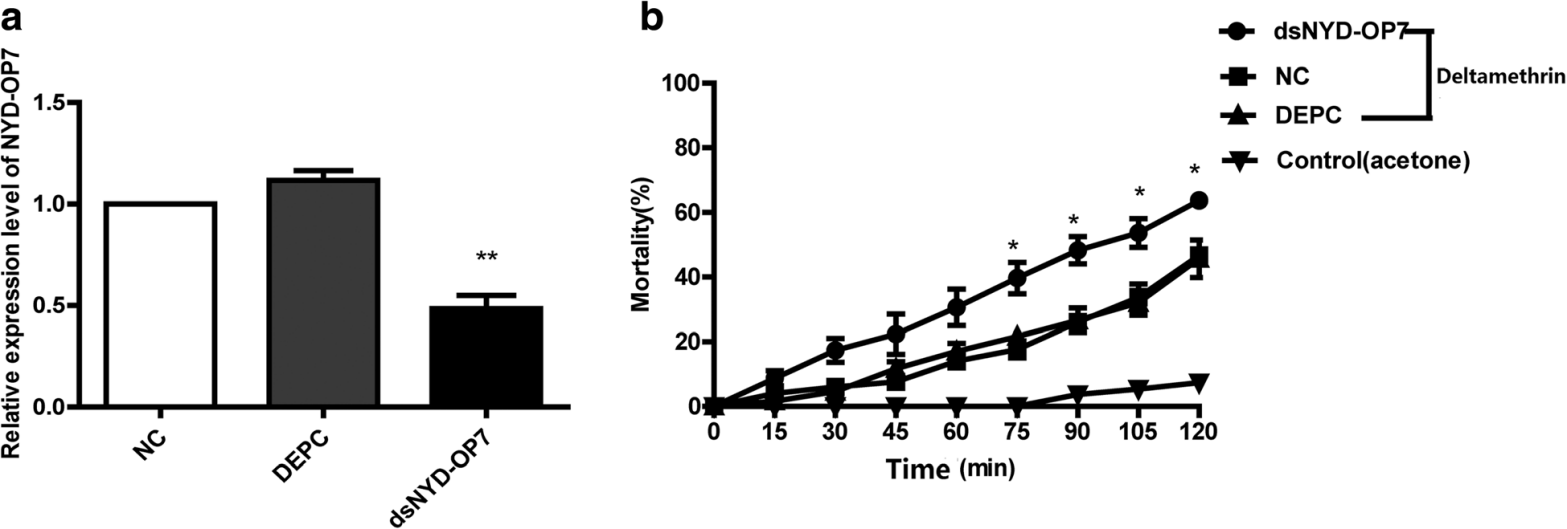
NYD-OP7 modulates deltamethrin resistance in mosquitoes. **a** Expression levels of *NYD-OP7* after microinjection of dsNYD-OP7. **b** Mortality of dsNYD-OP7-microinjected mosquitoes observed after 2 h of exposure to deltamethrin in CDC bottles

To identify the effects of *NYD-OP7* on deltamethrin resistance, we injected dsNYD-OP7 into the DR strain. qPCR showed that the knockdown efficiency of *NYD-OP7* was 45% when compared with the NC group (Fig. 2a). On the basis of the results of the CDC bottle bioassay, the group injected with dsNYD-OP7 showed a higher mortality rate compared to the NC group at 75, 90, 105 and 120 min, suggesting that knockdown of *NYD-OP7* can sensitize mosquitoes to deltamethrin (Fig. 2b).

**Fig. 3:**
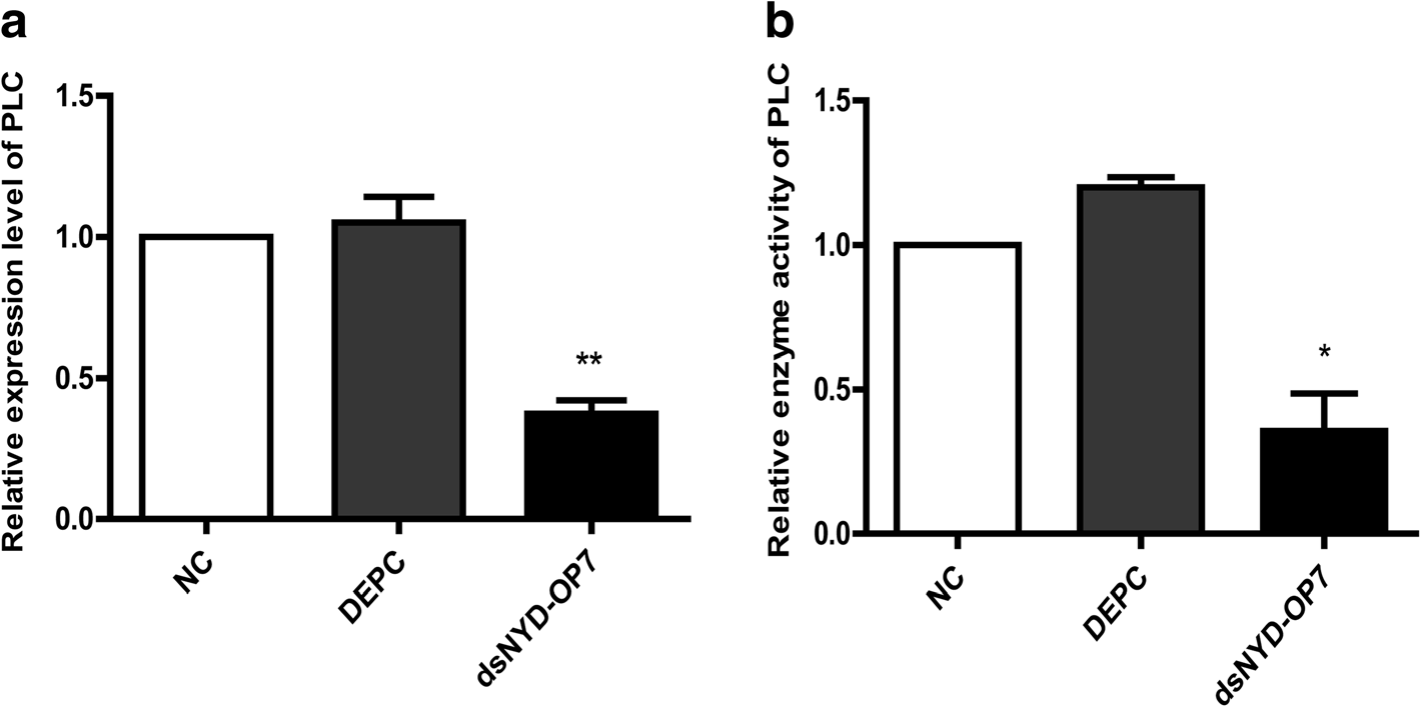
PLC is regulated by NYD-OP7. **a** Expression levels of PLC after injection of dsNYD-OP7. **b** Enzyme activity of PLC after injection of dsNYD-OP7

To clarify how *NYD-OP7* plays a role in deltamethrin resistance, we tested changes in the expression and enzyme activity of its downstream effector *PLC*. The expression and enzyme activity of *PLC* decreased by 52% and 65%, respectively, in the dsNYD-OP7 group when compared to the NC group (Fig. 3a, b).

**Fig. 4:**
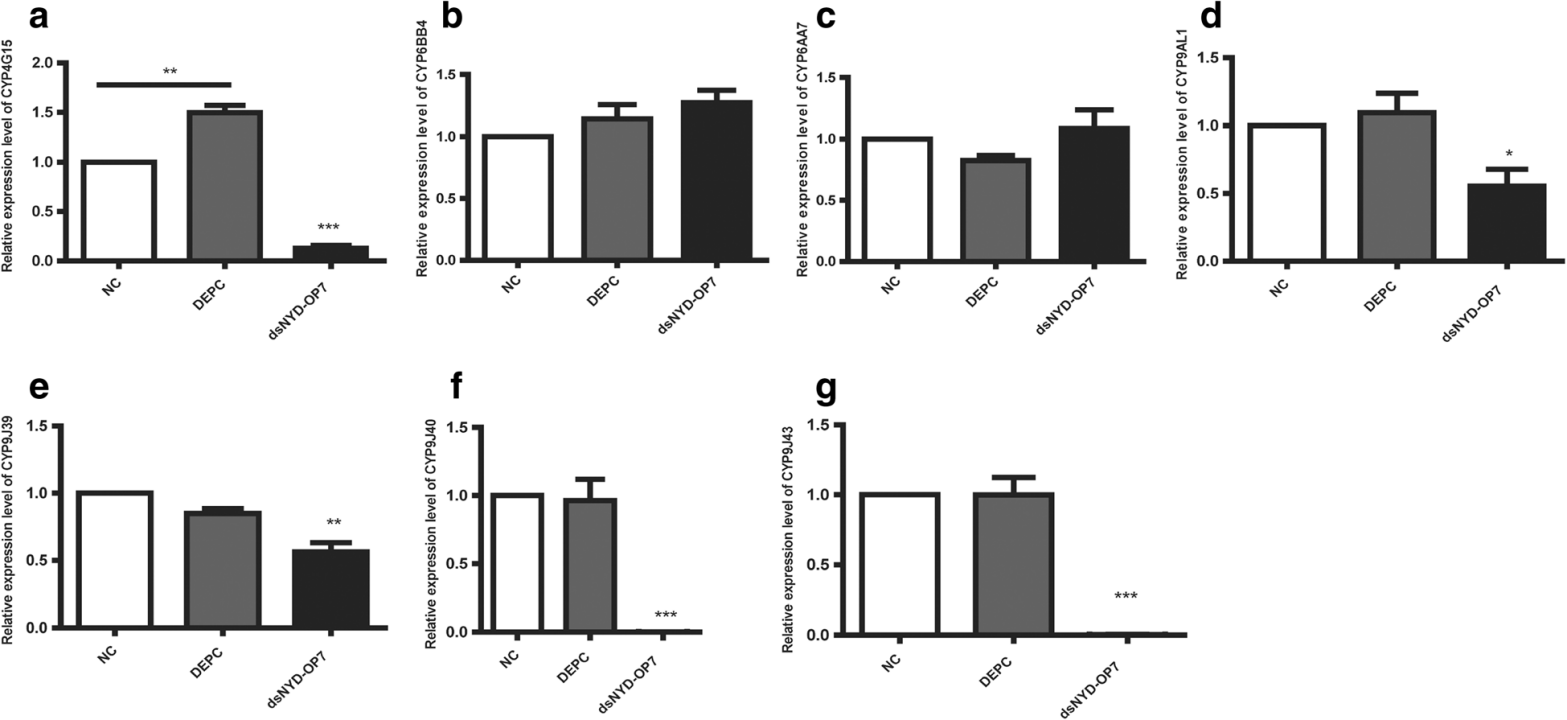
Expression levels of the P450 genes after injection of dsNYD-OP7. **a** Expression levels of *CYP4G15* after dsNYD-OP7 injection. **b** Expression levels of *CYP6BB4* after dsNYD-OP7 injection*.* **c** Expression levels of *CYP6AA7* after dsNYD-OP7 injection*.* **d** Expression levels of *CYP9AL1* after dsNYD-OP7 injection*.* **e** Expression levels of *CYP9J39* after dsNYD-OP7 injection. **f** Expression levels of *CYP9J40* after dsNYD-OP7 injection*.* **g** Expression levels of *CYP9J43* after dsNYD-OP7 injection

Since the GPCR signaling pathway can regulate P450 genes, which are strongly associated with the enhanced metabolic detoxification of insecticides, we also investigated the expression levels of a number of P450 genes. The qPCR results revealed a significant decrease in the expression levels of 5 P450 genes (*CYP4G15*, *CYP9AL1*, *CYP9J39*, *CYP9J40*, and *CYP9J43*) in the dsNYD-OP7 group when compared with the control (Fig. 4). Thus, our results indicate that the *NYD-OP7*/*PLC* regulatory signaling pathway, which governs P450 gene expression, may play a role in the regulation of resistance to deltamethrin in mosquitoes.

### Role of *PLC* in deltamethrin resistance

**Fig. 5:**
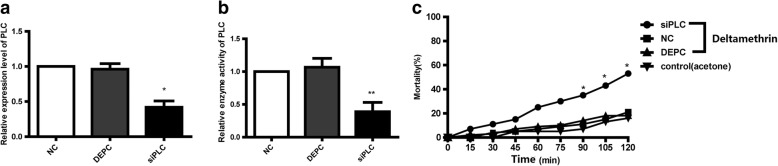
PLC modulates deltamethrin resistance in mosquitoes. **a** Expression levels of *PLC* after injection of siPLC. **b** Enzyme activity of PLC after injection of siPLC. **c** Mortality of siPLC-microinjected mosquitoes observed after 2 h of exposure to deltamethrin in CDC bottles

**Fig. 6:**
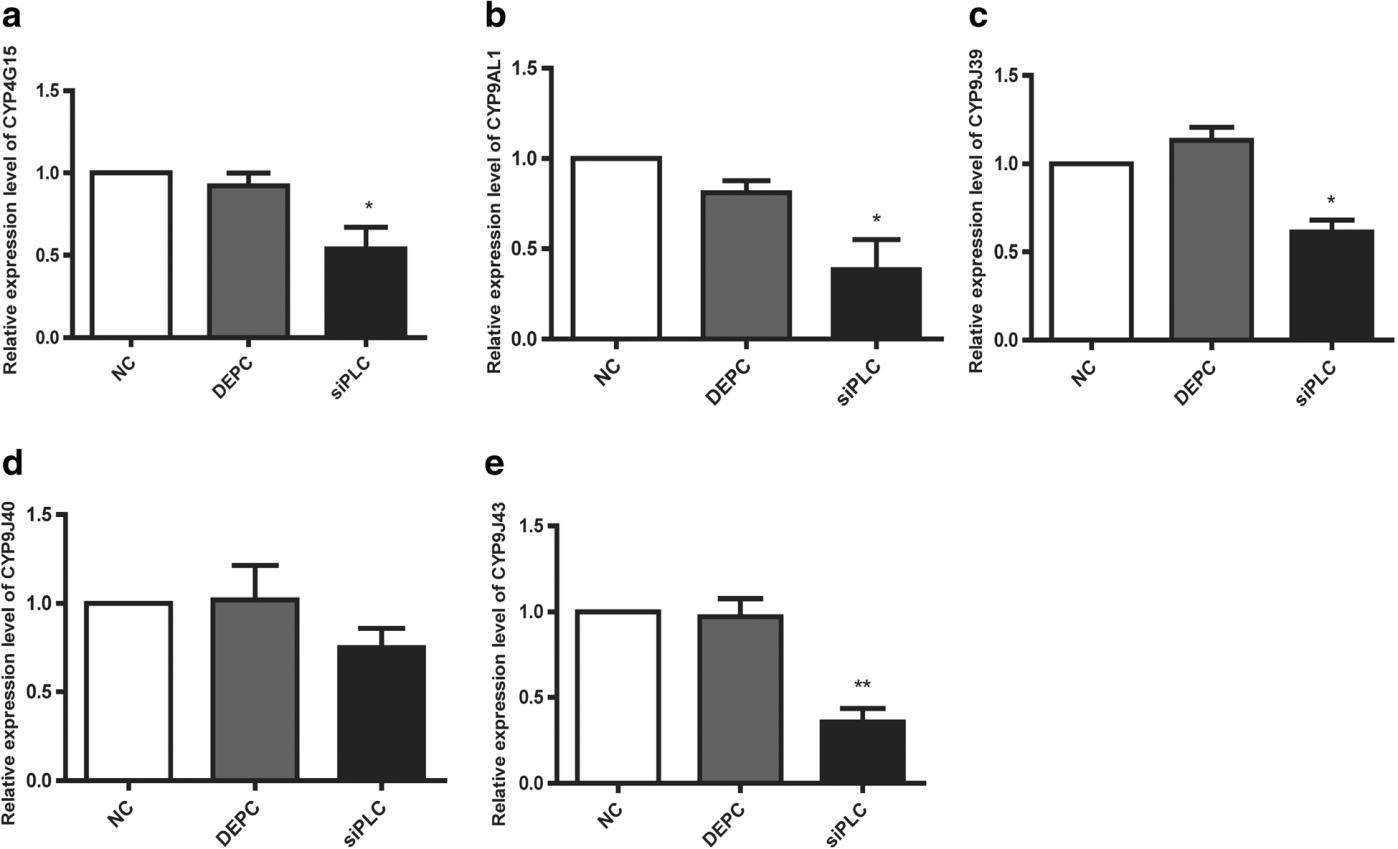
Expression levels of the P450 genes after injection of siPLC. **a** Expression levels of *CYP4G15* after siPLC injection. **b** Expression levels of *CYP9AL1* after siPLC injection*.* **c** Expression levels of *CYP9J39* after siPLC injection*.* **d** Expression levels of *CYP9J40* after siPLC injection*.* **e** Expression levels of *CYP9J43* after siPLC injection

To confirm our hypothesis that the *NYD-OP7*/*PLC* regulatory signaling pathway is involved in deltamethrin resistance, we performed the knockdown of *PLC*. The qPCR results showed that the knockdown efficiency of *PLC* was 59% when compared with the control group (Fig. 5a). Moreover, the enzymatic activity of PLC in the siPLC group decreased by 62% (Fig. 5b). The CDC bottle bioassay showed the mortality rate of the siPLC group increased significantly at 90, 105 and 120 min (Fig. 5c), functionally confirming the involvement of *PLC* in deltamethrin resistance. Further examination of P450 expression in the siPLC group revealed reduced expression of *CYP4G15*, *CYP9AL1*, *CYP9J39*, and *CYP9J43* (Fig. 6). However, inhibition of *PLC* had no effect on the expression of *CYP9J40*. Collectively, these results suggest that *NYD-OP7* and its downstream effector *PLC* contribute to deltamethrin resistance by regulating the expression of P450s.

## Discussion

Many diverse proteins with multiple functions are being identified. Opsin, which belong to a class of multifunctional proteins, has the roles of photoreceptor and the phospholipid flippase in human beings [30]. Our previous studies showed that *NYD-OP7*, which belongs to the GPCR family, may be involved in the development of insecticide resistance in *Cx. pipiens pallens* [34]*.* However, the exact mechanism is still unclear. Given the critical functions of the GPCR family in many essential biological and physiological processes, GPCRs have already been used as important targets for therapeutic interventions and provide a novel means of medical treatment for humans [38]. In insects, the GPCR signal transduction system has been shown to affect behavior, reproduction, osmoregulation, development and metabolism [39–41]. Moreover, recent studies have indicated that GPCRs could mediate insecticide resistance and provide opportunities for identifying new targets or vector control [32, 42]. Thus, a better understanding of the detailed role of *NYD-OP7* and its signaling pathway in insecticide resistance would be helpful for the management and the control of mosquito-borne diseases.

In invertebrates, *NYD-OP7* is a member of the Gq-coupled opsin subfamily, and an important step of the Gq-GPCR-mediated phototransduction pathway is activation of PLC [43]. In this study, knockdown of *NYD-OP7* resulted in a simultaneous reduction in the mRNA transcriptional levels and enzyme activity of PLC in *Cx. pipiens pallens*. Furthermore, knockdown of *NYD-OP7* or *PLC* resulted in decreased levels of resistance to deltamethrin in the mosquitoes. These findings suggest the potential role of the *NYD-OP7/PLC* signaling pathway in insecticide resistance in *Cx. pipiens pallens*.

P450s, which are phase I detoxification enzymes, are involved in the catabolism and anabolism of a diverse array of endogenous and xenobiotic compounds, such as insecticides [44]. The GPCR-mediated signaling pathway has been considered as a general regulatory factor for the expression of P450s in mosquitoes [32]. Thus, we hypothesized that the *NYD-OP7/PLC* signaling pathway may play a role in insecticide resistance through the regulation of the expression of P450 genes. To test our hypothesis, we examined the expression of P450 genes in dsNYD-OP7-injected mosquitoes and found that 5 P450 genes were downregulated after the knockdown of *NYD-OP7*. Knockdown of *PLC* decreased the expression of four P450 genes (one gene belongs to the CYP4 family and the other three genes belong to the CYP9 family). In insects, members of the CYP4 and CYP9 families have been associated with xenobiotic metabolism and play important roles in insecticide resistance [45–47]. Thus, our findings provide evidence that the *NYD-OP7/PLC* signaling pathway is involved in the development of resistance to deltamethrin in *Cx. pipiens pallens* by regulating the expression of the resistance-related P450 genes.

To date, the regulatory mechanisms of P450s are unclear. The expression of the resistance-related P450s is regulated by a variety of genetic and epigenetic factors. Our results revealed the role of the *NYD-OP7/PLC* signaling pathway in the regulation of P450 gene expression in mosquitoes. It is worth noting that *CYP9J40* was regulated only by *NYD-OP7*, and its expression was not influenced by *PLC*. One explanation could be that the GPCR-mediated signaling pathway and its regulation of P450 expression is a complex network. *PLC* is not the only downstream effector regulated by *NYD-OP7*. Other downstream components may interact with *NYD-OP7* to regulate other P450s.

In conclusion, our study demonstrates that the *NYD-OP7* and its downstream effector *PLC* signaling pathway contribute to deltamethrin resistance by regulating the expression of P450 genes in *Cx. pipiens pallens*, which provides novel insights into the role of the GPCR signaling pathway in insecticide resistance. Our results also offer a reference for further studies on the regulation of the P450 genes in mosquitoes and other insect species.

## Conclusions

In this study, we investigated the roles of *NYD-OP7*, which showed higher expression in the laboratory-selected DR strain of *Cx. pipiens pallens*. Depletion of *NYD-OP7* by RNAi not only increased the susceptibility of the mosquitoes to deltamethrin but also simultaneously repressed both activity and expression of the downstream effector molecule, PLC, and expression of several insecticide resistance-related P450 genes. Knockdown of *PLC* also sensitized the mosquitoes to deltamethrin and reduced the expression of the P450 genes. These results show that NYD-OP7 and its downstream effector PLC play significant roles in pyrethroid resistance.

## Additional files

Additional file 1: **Table S1.** Primers for *NYD-OP7*, *PLC*, and several P450 genes. (DOC 22 kb)

Additional file 2: **Table S2.** List of dsNYD-OP7, siPLC, and NC sequences. (DOCX 12 kb)

## Abbreviations

P450: Cytochrome P450
DR: Deltamethrin-resistant
DS: Deltamethrin-susceptible
PLC: Phospholipase C
GPCR: G-protein-coupled receptor
DDT: Dichlorodiphenyltrichloroethane
SSH: Suppression subtractive hybridization
qPCR: Quantitative real-time PCR
LC50: 50% lethal concentration
PBS: Phosphate-buffered saline
SDS-PAGE: Sodium dodecyl sulfate-polyacrylamide gel electrophoresis
NC: Negative control
DEPC: Diethyl pyrocarbonate
*OPN3*: *Opsin3*
PMSF: Phenylmethanesulfonyl fluoride
SEM: Standard error of the mean
ELISA: Enzyme-linked immunosorbent assay
