# Supplementary material for: NYD-OP7/PLC regulatory signaling pathway regulates deltamethrin resistance in Culex pipiens pallens (Diptera: Culicidae)
Source: Parasit Vectors. 2018 Jul 16;11:419. doi: 10.1186/s13071-018-3011-5 (PMC6048805; doi:10.1186/s13071-018-3011-5)
Supplement: Supplementary file 1 — Table S1. Primers for NYD-OP7, PLC, and several P450 genes. (DOC 22 kb) [file 13071_2018_3011_MOESM1_ESM.doc]

**Table S1**. Primers for *NYD-OP7*, *PLC*, and several P450 genes.

| Primers | Forward primer (5’ to 3’) | Reverse primer (5’ to 3’) |
| --- | --- | --- |
| NYD-OP7 | CGAAGAACAGATGCGTGAACA | GCCATGAACCACAGGGAAAT |
| PLC | CGGCAACTGTGGCTATCT | ACCGGCAATGACCTGTAC |
| β-actin | AGCGTGAACTGACGGCTCTTG | ACTCGTCGTACTCCTGCTTGG |
| CYP4G15 | CGAAGTGACGGTGGACAT | TCTTGATCTGACGGGAGTG |
| CYP6AA7 | AGCAGGAGTCTTCGGGTGA | CGTTAGTGCGGTTGAGGT |
| CYP6BB4 | ACCCACCTGCGGAAAGTCTA | AGTGTTGGCTGGGATGACG |
| CYP9J39 | GTTATTGGGTGGACTTGGG | CCCTGACTTTCACGAGCCTTA |
| CYP9J40 | TGACGAAGAGGCAGTTGAGAC | GAGACTTTCCGACACCACCA |
| CYP9J43 | ACCTCGGAGTGTTAGTCGTGTTTG | GGTATCGGTTTGTCGTGGAAG |
| CYP9AL1 | CTGGAGCCAAAGAAGTCG | TTCCCAACCAAACGCACA |
