# Supplementary material for: NYD-OP7/PLC regulatory signaling pathway regulates deltamethrin resistance in Culex pipiens pallens (Diptera: Culicidae)
Source: Parasit Vectors. 2018 Jul 16;11:419. doi: 10.1186/s13071-018-3011-5 (PMC6048805; doi:10.1186/s13071-018-3011-5)
Supplement: Supplementary file 2 — Table S2. List of dsNYD-OP7, siPLC, and NC sequences. (DOCX 12 kb) [file 13071_2018_3011_MOESM2_ESM.docx]

**Table S2**. List of dsNYD-OP7, siPLC, and NC sequences.

| Name | Sense (5’ to 3’) | Antisense (5’ to 3’) |
| --- | --- | --- |
| dsNYD-OP7 | CCTCGATCTGGACCATGACTTTGATTGCGTTTGACCGATACAATGTTATCGTGAAGGGTCTTGCCGCCAAGCCAATGACCAACAGTGGTGCTATGGTCAAGATCCTGCTCGTGTGGGCTTTCGCACTCTTCTGGACTCTGGCCCCATTCTTCGGATGGAACCGATATGTCCCAGAGGGTAACGTGACTGCCTGCGGAACTGGCTACCTGACCCAGACCTGGTTGAGCCGCTCGTACATAATCGTTTACGCCGTCTTCGTCTACTGGCTGCTCTGCTCACCATCATTTACTCGTACACGTTCATCCTGAAGGCT | AGCCTTCAGGATGAACGTGTACGAGTAAATGATGGTGAGCAGAGCAGCCAGTAGACGAAGACGGCGTAAACGATTATGTACGAGCGGCTCAACCAGGTCTGGGTCAGGTAGCCAGTTCCGCAGGCAGTCACGTTACCCTCTGGGACATATCGGTTCCATCCGAAGAATGGGGCCAGAGTCCAGAAGAGTGCGAAAGCCCACACGAGCAGGATCTTGACCATAGCACCACTGTTGGTCATTGGCTTGGCGGCAAGACCCTTCACGATAACATTGTATCGGTCAAACGCAATCAAAGTCATGGTCCAGATCGAGG |
| siPLC | CCACAACAUGUCGUCGUUUTT | AAACGACGACAUGUUGUGGTT |
| NC | UUCUCCGAACGUGUCACGUTT | ACGUGACACGUUCGGAGAATT |
